# Development and Validation of a Deep Learning System for Diagnosing Glaucoma Using Optical Coherence Tomography

**DOI:** 10.3390/jcm9072167

**Published:** 2020-07-09

**Authors:** Ko Eun Kim, Joon Mo Kim, Ji Eun Song, Changwon Kee, Jong Chul Han, Seung Hyup Hyun

**Affiliations:** 1 Department of Ophthalmology, Nowon Eulji Medical Center, Eulji University School of Medicine, Seoul 01830, Korea; csckek@gmail.com; 2 Department of Ophthalmology, Kangbuk Samsung Hospital, Sungkyunkwan University School of Medicine, Seoul 03181, Korea; kjoonmo1@gmail.com (J.M.K.); jj.song@samsung.com (J.E.S.); 3 Department of Ophthalmology, Samsung Medical Center, Sungkyunkwan University School of Medicine, Seoul 06351, Korea; ckee@skku.edu; 4 Institute of Biomedical Artificial Intelligence, SAIHST, Sungkyunkwan University, Seoul 06351, Korea; 5 Department of Nuclear Medicine, Medical AI Research Lab, Samsung Medical Center, Sungkyunkwan University School of Medicine, Seoul 06351, Korea

**Keywords:** deep learning system, glaucoma, spectral-domain optical coherence tomography, diagnostic ability, retinal nerve fiber layer, ganglion cell–inner plexiform layer

## Abstract

This study aimed to develop and validate a deep learning system for diagnosing glaucoma using optical coherence tomography (OCT). A training set of 1822 eyes (332 control, 1490 glaucoma) with 7288 OCT images, an internal validation set of 425 eyes (104 control, 321 glaucoma) with 1700 images, and an external validation set of 355 eyes (108 control, 247 glaucoma) with 1420 images were included. Deviation and thickness maps of retinal nerve fiber layer (RNFL) and ganglion cell–inner plexiform layer (GCIPL) analyses were used to develop the deep learning system for glaucoma diagnosis based on the visual geometry group deep convolutional neural network (VGG-19) model. The diagnostic abilities of deep learning models using different OCT maps were evaluated, and the best model was compared with the diagnostic results produced by two glaucoma specialists. The glaucoma-diagnostic ability was highest when the deep learning system used the RNFL thickness map alone (area under the receiver operating characteristic curve (AUROC) 0.987), followed by the RNFL deviation map (AUROC 0.974), the GCIPL thickness map (AUROC 0.966), and the GCIPL deviation map (AUROC 0.903). Among combination sets, use of the RNFL and GCIPL deviation map showed the highest diagnostic ability, showing similar results when tested via an external validation dataset. The inclusion of the axial length did not significantly affect the diagnostic performance of the deep learning system. The location of glaucomatous damage showed generally high level of agreement between the heatmap and the diagnosis of glaucoma specialists, with 90.0% agreement when using the RNFL thickness map and 88.0% when using the GCIPL thickness map. In conclusion, our deep learning system showed high glaucoma-diagnostic abilities using OCT thickness and deviation maps. It also showed detection patterns similar to those of glaucoma specialists, showing promising results for future clinical application as an interpretable computer-aided diagnosis.

## 1. Introduction

Artificial intelligence (AI) is a branch of computer science that seeks to simulate intelligent human behavior in computers [1,2]. Deep learning, a state-of-the-art technique enabled by advancements in graphics processing units and processing power, has revolutionized the use of AI since 2010 [3]. The resulting improvements in computer programming have allowed AI to become integral to medical decision-making and have encouraged researchers to develop deep learning algorithms for diagnosing vision-threatening ophthalmic diseases including glaucoma. Since accurate screening and proper surveillance is essential to decrease the socioeconomic burden of patients suffering from glaucoma, clinicians have developed and expanded the application of deep learning systems for diagnosing glaucoma using various datasets and algorithms [4].

Spectral-domain optical coherence tomography (SD-OCT), which provides reproducible, objective, and quantitative retinal nerve fiber layer (RNFL) and ganglion cell–inner plexiform layer (GCIPL) thickness measurements, is currently one of the most commonly used diagnostic tools for glaucoma [5,6,7]. Along with providing actual thickness measurements, it also offers thickness maps and deviation maps demonstrating diagnostic classification in comparison with a normative database, which aids clinicians in the identification of glaucomatous damage [8]. However, it has limitations preventing its use as a solitary glaucoma screening system, namely artifacts [9], segmentation errors [10], and the possibility of both false-positive and false-negative results [11]. Thus, the development of a well-trained deep learning system that can provide an interpretable computer-aided glaucoma diagnosis using OCT images would be useful to overcome these limitations and to allow glaucoma screenings of large populations.

Recently, deep learning with convolutional neural networks (CNN) has been widely used for pattern recognition and classification of medical images [3,12]. A number of studies reported successful diagnostic performance of CNNs for detection of glaucoma based on fundus photographs [13,14,15]. However, validation of deep learning systems using various OCT maps has not been explored fully. SD-OCT can reveal glaucomatous damage in the optic nerve head (ONH) and peripapillary area as well as in the macular area, and thus, the inclusion of such data is promising to improve the glaucoma diagnostic ability of deep learning systems. Furthermore, the SD-OCT results represent an abundant dataset that can easily train a deep learning system. Therefore, we developed and validated a deep learning system for glaucoma diagnosis using OCT deviation and thickness maps of RNFL and GCIPL analyses. We also evaluated heatmaps to visualize the diagnostic pattern of our deep learning system.

## 2. Materials and Methods

RNFL and GCIPL images acquired by SD-OCT (Cirrus SD-OCT; Carl Zeiss Meditec Inc., Dublin, CA, USA) were consecutively collected between 2015 and 2019 from the glaucoma clinics of Samsung Medical Center and Kangbuk Samsung Hospital. The present study was approved by the Institutional Review Boards of Samsung Medical Center and Kangbuk Samsung Hospital, and adhered to the tenets of the Declaration of Helsinki.

### 2.1. Subjects—Training, Internal Validation, and External Validation Datasets

Subjects who visited the glaucoma clinic of Samsung Medical Center between 2015 and 2019 were divided into a training set and an internal validation set of glaucoma and control groups using random sampling. To build the training set, four OCT images (thickness and deviation maps each of RNFL and GCIPL analyses) were obtained from each of 1490 eyes of 967 primary open-angle glaucoma (POAG) patients and 332 eyes of 245 healthy subjects, giving a total of 7288 OCT training images. OCT images were acquired for the internal and external validation sets in the same manner used to acquire those of the training set. The internal validation dataset consisted of images from 321 eyes of 299 POAG patients and 104 eyes of 98 healthy subjects, giving 1700 OCT internal validation images. Images taken from subjects at Kangbuk Samsung Hospital constituted the external validation set; these were taken from 247 eyes of 147 POAG patients and 108 eyes of 69 healthy subjects, giving 1420 OCT external validation images.

Each subject underwent a complete ophthalmic examination including intraocular pressure measurement by Goldmann applanation tonometry, gonioscopy, biomicroscopy, and fundus examination by glaucoma specialists. They also underwent automatic refraction (KR-800A; Topcon Medical Systems Inc., Oakland, NJ, USA), stereo optic disc and red-free RNFL photography (TRC-50DX; Topcon Medical Systems Inc., Oakland, NJ, USA), central corneal thickness measurement by ultrasound pachymetry (Tomey SP-3000; Tomey Ltd., Nagoya, Japan), axial length measurement (IOL Master; Carl Zeiss Meditec Inc., Dublin, CA, USA), SD-OCT examination, and standard automated perimetry using the Swedish interactive threshold algorithm with the 30-2 standard program (Humphry Field Analyzer II; Carl Zeiss Meditec Inc., Dublin, CA, USA).

For POAG diagnosis, the inclusion criteria were eyes with the presence of typical glaucomatous changes in the ONH, including rim notching, thinning, and/or wedge-shaped RNFL defect or diffuse RNFL atrophy; open angle on gonioscopy; no history of retinal disease, optic neuropathy, or systemic/neurologic disease that could affect RNFL/GCIPL OCT scans or visual field (VF) tests; and glaucomatous VF defect, as confirmed by at least two consecutive VF examinations. Reliable VF tests were defined as those having false-negative rates less than 15%, false-positive rates less than 15%, and fixation losses less than 20%. Glaucomatous defects were assigned for VF tests showing any cluster of three points or more with *p* < 0.05 on the pattern deviation map in at least one hemifield, including one point or more with *p* < 0.01; a pattern standard deviation of *p* < 0.05; or glaucoma hemifield test results outside the normal limits [16]. Glaucomatous eyes were further classified by severity into VF severity grades [17] of early (MD > −6 decibels (dB)), moderate (−12 dB < MD ≤ −6 dB), and advanced glaucoma (MD ≤ −12 dB).

For healthy controls, the inclusion criteria were eyes with best corrected visual acuity ≥20/25; open angle on gonioscopy; absence of glaucomatous optic neuropathy and RNFL defect; no history of elevated intraocular pressure; no history of intraocular surgery except simple cataract surgery; no history of retinal disease or any kinds of optic neuropathy that could affect OCT scans; and normal VFs.

### 2.2. Glaucoma Diagnosis by Glaucoma Specialists

Initial diagnosis (glaucoma or control) was made by two experienced glaucoma specialists (C.K., J.M.K.) independently of each other and served as a reference standard. Each diagnosis was determined based upon observation of disc and RNFL photographs and VFs without knowledge of the patient’s clinical information. Discrepancies were resolved by consensus through discussion; if no consensus was reached, the case was excluded from the final dataset. Moreover, for representative comparison of the diagnostic ability between the deep learning system and glaucoma specialists, two additional experienced glaucoma specialists (J.C.H., K.E.K.) independently diagnosed each case in a blind manner using the RNFL thickness map, the same image dataset used to test the deep learning system.

### 2.3. Spectral-Domain Optical Coherence Tomography Examination

The SD-OCT examination was performed after pupil dilation. One macular scan focusing on the fovea and one peripapillary scan focusing on the optic disc (all 200 × 200 cube protocol) were acquired for every subject. Only OCT images with scans of good quality were included for analysis. Data with segmentation errors, motion artifacts due to eye movements or involuntary blinking, fixation error, or signal strength index <7 were excluded from analysis. OCT images with severe resolution reduction or significant artifacts were also excluded. Among the various OCT maps, deviation and thickness maps from ONH/RNFL analysis and macular ganglion cell analysis were included in the dataset; left eye images were mirrored to a right eye orientation.

The methods have been described in detail previously [8]. Briefly, an optic disc cube obtained from a three-dimensional dataset that covers a 6 mm^2^ area is centered on the optic disc. After generating an RNFL thickness map from the cube dataset, the software automatically determines the center of the disc and then positions a calculation circle 3.46 mm in diameter from the cube dataset for RNFL thickness measurement. The ganglion cell analysis algorithm detects and measures macular GCIPL thickness within an annulus of inner vertical and horizontal diameters of 1 and 1.2 mm, respectively, and outer vertical and horizontal diameters of 4 and 4.8 mm, respectively. Based on these automatic measurement data of RNFL and macular GCIPL thickness, SD-OCT provides a thickness map and a deviation map. For both RNFL and GCIPL deviation maps, the areas appear as yellow or red to represent thicknesses less than the lower 5% or 1% in comparison with age-matched normative data, respectively. Uncolored areas indicate RNFL or GCIPL thickness within the normal range.

### 2.4. Deep Learning Framework

**Figure 1:**
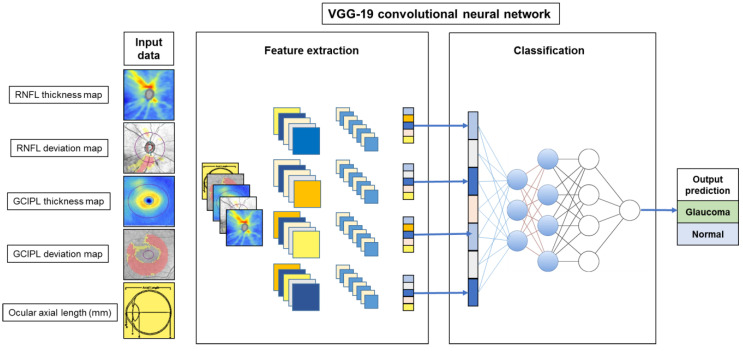
Schematic overview of the proposed deep learning model of convolutional neural network (CNN)-based visual geometry group deep (VGG-19) architecture. The input data were a stack of two-dimensional images (176 × 176 pixel) composed of four optical coherence tomography images (thickness and deviation maps of retinal nerve fiber layer (RNFL) and ganglion cell–inner plexiform layer (GCIPL) analyses) and a matrix image with an ocular axial length value.

We used a CNN–based framework for classification in this study; the architecture of the deep learning model is presented in Figure 1. The visual geometry group deep CNN (VGG-19), the state-of-the-art image classification task proposed by Simonyan and Zisserman [18], was our base deep learning model architecture for glaucoma diagnosis using OCT maps. This VGG-19 network consists of 19 layers that are grouped into five stacks of convolutional layers with 3 × 3 kernels and maximum pooling. A stack of convolutional layers is followed by three fully connected layers with 4096, 4096, and 1000 channels, respectively. The convolutional neural layers were used to extract local feature representations to the next layer, while the fully connected layers were used to predict the classification (glaucoma/normal) result. It has been widely adopted to solve image classification tasks in both medical and general fields and has shown excellent results [12].

We trained the network from scratch with the input of a stack of two-dimensional images (176 × 176 pixel) composed of four OCT images (thickness and deviation maps of RNFL and GCIPL analyses) and a matrix image with an ocular axial length value. For comparison of the diagnostic performance of combination models, the number of input images ranged from 1 to 5. By performing convolution and fully connected layer training, the output of the softmax layer represents the probability of glaucoma diagnosis. For the training model, we used the stochastic gradient descent optimizer with a momentum of 0.9, a learning rate of 0.01, a batch-size of 8, and a maximum learning epoch of 100. During training, we snapshot the model every 1 epoch and selected one with the highest validation accuracy as the final model. This framework was implemented in PyTorch 1.2 on Windows 10 with Nvidia GTX 1080Ti graphics processing unit support.

### 2.5. Heatmap Analysis

As a visual aid to help explain the results produced by the deep learning–based diagnosis system, heatmaps highlighting the important regions in each OCT image for predicting glaucoma were generated using the Gradient-weighted Class Activation Mapping (Grad-CAM) algorithm [19]. Heatmaps from the internal validation dataset were reviewed by glaucoma specialists to validate the model. Two experienced glaucoma specialists (J.C.H., K.E.K.) independently evaluated the locations of RNFL and macular GCIPL defects on original OCT maps in a masked manner. Discrepancies between the two specialists’ findings were resolved through discussion. Agreements upon defect locations between glaucoma specialists and heatmaps produced based upon the deep learning model were scored as either excellent, partial, or no agreement.

### 2.6. Statistical Analyses

The baseline characteristics of glaucomatous eyes and controls were compared using the independent *t*-test for continuous variables and the chi-square test for categorical variables. Comparison of baseline characteristics between eyes of different glaucoma severities (early, moderate, and severe) was performed using the one-way analysis of variance test for continuous variables and the chi-square test for categorical variables. The Tukey test was used for post hoc analysis. Sensitivities and specificities were assessed to analyze the diagnostic ability of glaucoma specialists when using the RNFL thickness map. The McNemar test was used to compare the automated diagnosis of the deep learning system against the reference standard and against glaucoma specialists’ diagnosis of OCT maps. For comprehensive evaluation of the diagnostic performance of the trained deep learning model, it was tested with the independent datasets (i.e., the internal and external validation datasets), and the area under the receiver operating characteristic curve (AUROC) as well as 95% confidence intervals (CIs) and sensitivities at the fixed specificities of 80% and 90% were calculated. The DeLong test was used to test the statistical significance of the diagnostic performance difference (represented as AUROC) between any two parameters [20]. The AUROCs of different variables were compared using MedCalc software version 12.0 (MedCalc Statistical Software, Marakierke, Belgium). Other statistical analyses were performed using Statistical Package for the Social Sciences version 21.0 for Windows (IBM Corp., Armonk, NY, USA). Statistical significance was defined as *p* < 0.05.

## 3. Results

Table 1 presents baseline characteristics of included subjects. In the training dataset, the glaucoma group had significantly higher mean age, lower RNFL and GCIPL thicknesses, and lower mean deviation values compared to the control group (all *p* ≤ 0.001). Similar results were also found in respective group comparisons from the internal and external validation datasets. Table 2 summarizes the characteristics of subjects from the internal validation set according to glaucoma severity. No significant difference was found among them in age, gender, and axial length. However, lower RNFL and GCIPL thicknesses and lower VF MD values were found in eyes with more severe degrees of glaucomatous damage (all *p* < 0.001).

Comparison between glaucomatous eyes with different severities was performed using the one-way analysis of variance test (for continuous variables) and the chi-square test (for categorical variables).

The Tukey test was used for post hoc analysis. In the post hoc analysis, *p* < 0.05 was regarded as the significance criterion.

### 3.1. Diagnostic Ability of Deep Learning Systems Using OCT Maps

The AUROC of the deep learning system using OCT maps was high, ranging from 0.903 to 0.987 (Table 3). The diagnostic ability of the deep learning system based on single OCT maps was the highest when using the RNFL thickness map (AUROC 0.987), followed by the RNFL deviation map (AUROC 0.974), the GCIPL thickness map (AUROC 0.966), and the GCIPL deviation map (AUROC 0.903). For deep learning systems using combinations of OCT maps, the combination of RNFL and GCIPL deviation maps demonstrated the highest diagnostic performance (AUROC 0.979), followed by that of all four OCT maps (AUROC 0.977), and that of RNFL and GCIPL thickness maps (AUROC 0.964).

The diagnostic ability of the deep learning system using the RNFL thickness map did not differ statistically significantly from that using the RNFL deviation map (*p* = 0.10), but it significantly outperformed that using the GCIPL thickness map (*p* = 0.022). It also showed no significant difference in diagnostic performance compared to the deep learning system based on the combination of RNFL and GCIPL deviation maps (*p* = 0.24) but was significantly better than the deep learning system based on combination of RNFL and GCIPL thickness maps (*p* = 0.033) and that based on all four OCT maps (*p* = 0.013). Similar results were found when tested by using the external validation dataset (Table S1).

The glaucoma diagnostic performance of the deep learning system was compared among different glaucoma severity subgroups using the internal validation dataset (Table 4). Despite its overall excellent ability, the diagnostic ability increased with increasing disease severity. In early glaucoma, use of the RNFL thickness map alone showed the highest diagnostic ability (AUROC 0.974), followed by the combination of RNFL and GCIPL deviation maps (AUROC 0.965). In moderate and severe glaucoma, most of the maps except the GCIPL deviation map (AUROC 0.935) showed excellent diagnostic performance, with AUROC ranging between 0.965 and 0.999.

**Figure 2:**
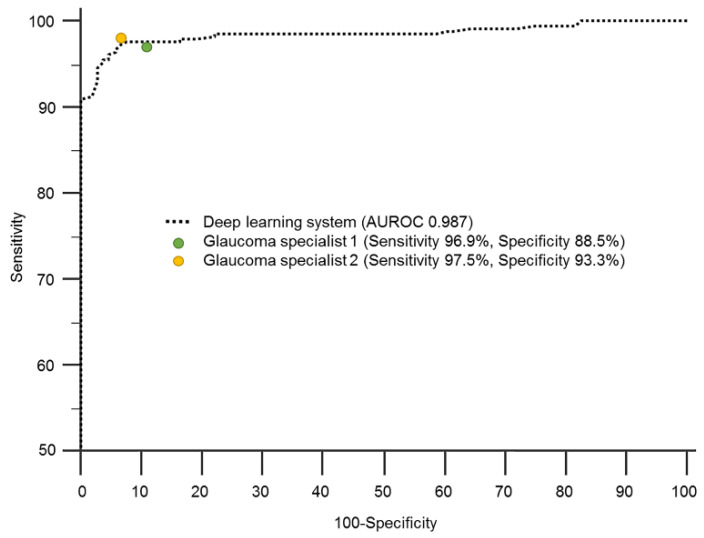
Area under the receiver operating characteristic curve (AUROC) of a deep learning system for glaucoma diagnosis based on a retinal nerve fiber layer thickness map of spectral-domain optical coherence tomography, in comparison with that of glaucoma specialists.

As the deep learning system using only the RNFL thickness map showed the best diagnostic performance, its diagnostic ability was compared with that of two glaucoma specialists for representative comparison. The sensitivities of the two glaucoma specialists were 96.9% and 97.5% and their specificities were 88.5% and 93.3%, respectively, showing similar results to the deep learning system (Figure 2).

### 3.2. Heatmap Analysis

**Figure 3:**
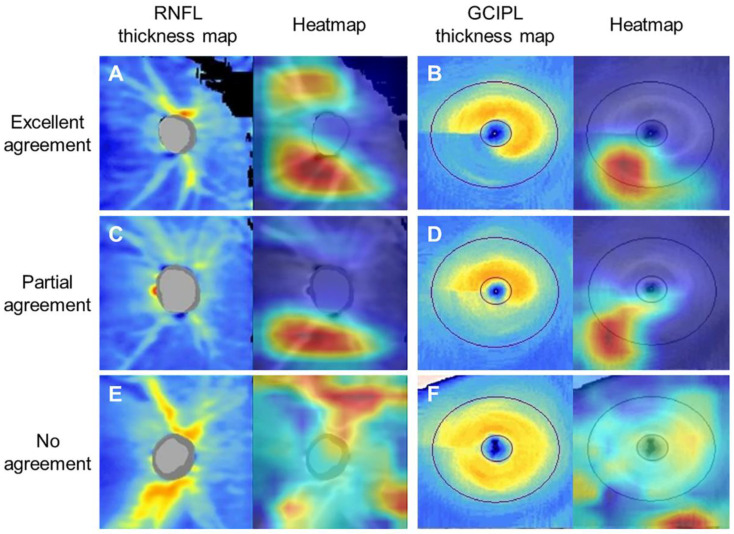
Heatmaps highlighting the region of high probability for glaucoma diagnosis were generated to validate the deep learning system using retinal nerve fiber layer (RNFL) and ganglion cell–inner plexiform layer (GCIPL) thickness maps of spectral-domain optical coherence tomography. Images are representative RNFL and GCIPL thickness maps and corresponding heatmaps for which the deep learning system produce predictions resulting in (**A**,**B**) excellent, (**C**,**D**) partial, and (**E**,**F**) no agreement with glaucoma specialists on RNFL and GCIPL defect locations. A. Glaucoma specialists confirmed superotemporal and inferotemporal RNFL defect from the RNFL thickness map; the heatmap showed that the deep learning system detected the same lesion, showing high agreement between them. B. Glaucoma specialists and the heatmap agreed on an inferotemporal GCIPL defect. C. Superotemporal and inferotemporal RNFL defects were confirmed by glaucoma specialists, but only the inferotemporal lesion was demonstrated on the heatmap, thus showing partial agreement. D. A whole inferior GCIPL defect was confirmed by a glaucoma specialist, but only part of the lesion was shown on the heatmap. E. Discrepancy between the colored lesion on the heatmap and the thin, superotemporal RNFL defect detected by glaucoma specialists, showing no agreement. F. A thin, inferotemporal GCIPL defect was confirmed by glaucoma specialists, but the heatmap showed an irrelevant lesion.

For visualization of our deep learning–based diagnostic system and to confirm the areas contributing most to the diagnosis, heatmaps were generated by using the Grad-CAM algorithm. Specifically, heatmaps generated from the deep learning system using the RNFL and GCIPL thickness maps were evaluated in particular, which showed the highest AUROC in the deep learning system using RNFL and GCIPL maps. Analysis was conducted on the agreement regarding the location of glaucomatous damage on RNFL and GCIPL thickness maps between the heatmaps from the deep learning system and those from glaucoma specialists (Figure 3). The location of RNFL damage on the heatmap produced using the RNFL thickness map generally agreed with that indicated by glaucoma specialists, with 90.0%, 8.0%, and 2.0% of excellent, partial, and no agreement, respectively. The location of GCIPL damage indicated on the heatmap produced using the GCIPL thickness map also showed an excellent level of agreement with that indicated by glaucoma specialists, with 88.0%, 6.4%, and 5.6% of excellent, partial, and no agreement, respectively.

## 4. Discussion

The SD-OCT is currently one of the most commonly used ancillary tests to diagnose glaucoma [5,6,7,8,21]. Despite its high sensitivity and specificity [8], OCT maps have limitations in intelligently elucidating the final diagnosis, and thus, clinicians have to make their own interpretations. However, with the help of an extensively trained deep learning system, a final diagnosis can be automatically generated. Such diagnoses could help ophthalmologists with rapid clinical decision making and furthermore facilitate glaucoma screening. In light of these possibilities, we investigated and validated the diagnostic ability of a deep learning–based glaucoma diagnostic system using VGG-19 and various OCT maps. The strength of our study is that we used all the currently available RNFL and GCIPL deviation and thickness maps from glaucomatous eyes of various disease severities and provided heatmap analysis to visualize the diagnostic patterns of the deep learning system.

The deep learning system showed great potential to enhance glaucoma diagnosis as confirmed in a number of studies. Along with the excellent glaucoma diagnostic ability of deep learning systems when using fundus photographs [13,14,15,22], deep learning systems using OCT for classification of glaucomatous change in the peripapillary and macular areas also have shown good results [23,24,25,26,27,28]. Deep learning systems have been trained to automatically diagnose glaucoma based on OCT measurements including RNFL/GCIPL thickness, minimum rim width relative to Bruch’s membrane opening measurements, or ONH volume scans, demonstrating sensitivity and specificity over 90.0%. Different from previous studies, the present study developed and validated a deep learning system trained on all of the commonly used OCT maps, namely the thickness and deviation maps of RNFL and GCIPL analyses. Among the four OCT maps, use of the RNFL thickness map alone yielded the highest AUROC of 0.987, and the use of the macular GCIPL thickness map ranked second. The thickness map may be more accurate than the deviation map as an information provider as it presents real thickness change in an easily identified, colored pattern. The diagnostic performance of deep learning systems using deviation maps can be limited, particularly in cases showing false-positive diagnostic classification, which can train the neural network to imitate the original errors on deviation maps. Interestingly, the diagnostic ability of a deep learning system based on RNFL maps was generally better than that of a deep learning system based on GCIPL maps. This is probably in line with previous results that the diagnostic ability of RNFL thickness measurements or RNFL maps was relatively better than that of GCIPL thickness measurements of GCIPL maps, regardless of statistical significance [29,30,31]. Nonetheless, the overall diagnostic performance was high for single maps and combinations of thickness and deviation maps, suggesting that use of the deep learning system with various OCT maps has potential as a valuable diagnostic aid in glaucoma.

One major limitation of OCT is that its results are affected by the patients’ myopic degree (represented by axial length or refractive measurements) [11,32,33], and thus, we additionally evaluated whether providing the deep learning system with axial length would alter its diagnostic performance. Our hypothesis was that if the deep learning system could identify the difference in OCT findings between myopic and non-myopic eyes using axial length as a diagnostic cue, it would lower the false-positive rates in myopic eyes, leading to increased specificity. However, adding axial length did not significantly improve the diagnostic ability of our deep learning system in any case. We speculate that the observed results occurred for the following reasons. First, although our dataset included a large proportion of myopic eyes, the proportion of extremely highly myopic eyes having eccentric, ungradable OCT findings was low, and thus the deep learning system did not have difficulty in diagnosing glaucoma in myopic eyes. Second, the deep learning system may already have acquired other unknown algorithms that can differentiate myopic eyes with glaucoma from healthy myopic eyes. In such a case, axial length might provide no additional benefit. Finally, due to the high level of diagnostic performance of the deep learning system using only a single OCT thickness map, the effect of axial length may have been too subtle to elicit a statistically significant difference. These results may indicate that, even for myopic eyes, the deep learning system can be trained to achieve a high standard of glaucoma diagnostic ability by using only OCT maps.

Ophthalmologists rely on pattern recognition of changes in the ONH and the peripapillary area to diagnose glaucoma. This dependence on visualization of glaucomatous damage is well suited to benefit from combinations of glaucoma diagnostic devices and deep learning systems. Various deep learning algorithms, including ResNet, VGGNet, AlexNet, and GoogLeNet have been developed and improved over time for classification of medical images [2,12]. These have been combined with various types of OCT data for improvement in glaucoma diagnosis. Asaoka et al. [28] developed a CNN classifier to diagnose early glaucoma using RNFL and macular ganglion cell complex thickness measurements from RNFL and macular OCT images with an AUROC of 0.937. They presented outstanding results by transfer learning using a large pretraining dataset. The ResNet was used by Ran et al. [26] for glaucoma detection using OCT volume scans of the optic nerve head. They also reported high diagnostic performance in three external validation datasets, with AUROCs of 0.893–0.897 (sensitivities of 78–90% and specificities of 79–86%). Direct comparison of our results with prior studies is difficult due to different patient characteristics, validation methods, diagnostic modalities, and type of images used as input data. Nonetheless, we initially conducted a comparison analysis between VGG-19 and ResNet-34 models. Despite no significant difference in diagnostic performance between VGG-19 (AUROC 0.987, 95% CI 0.971–0.995) and ResNet-34 (AUROC 0.978, 95% CI 0.959–0.990; *p* = 0.076) when using the RNFL thickness map, the VGG-19 showed diagnostic patterns more compatible with those of glaucoma specialists than did the ResNet-34 in heatmap analysis. Consequently, we chose the VGG-19, a well-known CNN in biomedical image analysis, for its strong advantages in large-scale image processing and its high speed and outstanding performance. Different from fundus photographs, OCT maps have smaller number of pixels, which might have required more sophisticated processing. The VGG-19, based on our results, showed that it can provide reliable predictions in glaucoma diagnosis even when using OCT images.

It is well known that the diagnostic sensitivity of OCT increases as the glaucoma stages advance [34]. The deep learning system showed a similar tendency, having slightly lower sensitivity for eyes with early glaucoma compared to that for those in more severe stages. In addition, cases showing no agreement between glaucoma specialists and the deep learning system were found mostly in eyes with early glaucoma. Patients with early glaucomatous damage can be difficult to diagnose using only a single OCT image without other relevant clinical information, even for glaucoma specialists. The deep learning system using OCT maps, in general, showed good diagnostic ability even in early stages. However, the deep learning system using the GCIPL thickness map showed a statistically significantly decreased diagnostic ability for the early stage. Use of the GCIPL deviation map showed no significant difference in diagnostic ability between stages, but this was attributed to relatively low diagnostic ability throughout all stages. Despite the generally high diagnostic performance of the deep learning system even for early glaucoma, further technical development is required to improve the diagnostic ability of the deep learning system using GCIPL maps for the early stage.

The deep learning system can learn autonomously through training, but there is still a need for ophthalmologists to supervise and confirm its detection pattern to allow its general application in clinics. In this regard, we evaluated heatmap patterns for better understanding of the region of interest detected by the deep learning system. Numerous studies have reported that glaucomatous peripapillary RNFL damage mainly occurs in the superotemporal and inferotemporal regions, and the same applies for macular GCIPL damage [35]. The deep learning system using OCT thickness maps detected similar regions that were recognized by glaucoma experts with high levels of agreement. Partial or no diagnostic agreement existed between them, but overall the deep learning system mostly indicated the correct location of glaucomatous damage. The present study shows that if the interpretable deep learning system–aided OCT can demonstrate regions with high probability of glaucoma as a pre-interpreter, it will be able to reduce clinicians’ burden in busy clinics, and furthermore will be beneficial in detecting progressive change.

Several limitations should be considered when interpreting the present study. First, although we had two glaucoma specialists (C.K. and J.M.K.) to confirm the diagnosis of subjects from the training and validation sets, non-glaucomatous eyes could have been included in the dataset. However, as two separate glaucoma specialists (J.C.H. and K.E.K.) additionally evaluated the OCT images, we believe that this likely minimized the possibility of such a problem. Second, to support the generalizability of our deep learning system, we evaluated its diagnostic performance on an external validation set received from Kangbuk Samsung Hospital. Nevertheless, all OCT images were only from clinic-based samples of subjects with Asian ethnicities, and thus further investigation with a large number of population-based samples including diverse ethnicities is needed to validate our results. Finally, only gradable, good-quality OCT images were included in the present study, which may be limited in reflecting real clinical settings. Therefore, further training and testing with both gradable and ungradable OCT images should be incorporated into our deep learning–based diagnostic system to allow broader application.

In conclusion, the deep learning system using VGG-19 showed good glaucoma diagnostic ability when using deviation and thickness maps of RNFL and GCIPL analyses. Deep learning systems using various OCT maps have great potential to be used as a glaucoma diagnostic aid. Our findings may have valuable implications for establishing the computer-aided automatic interpretation of OCT data to serve as a good clinical decision-support tool.

## Figures and Tables

**Table 1:** Baseline characteristics of subjects included in the training, internal validation, and external validation datasets.

	Training Dataset		Internal Validation Dataset		External Validation Dataset	
	Control (*n* = 332)	Glaucoma (*n* = 1490)	Control (*n* = 104)	Glaucoma (*n* = 321)	Control (*n* = 108)	Glaucoma (*n* = 247)
Age (years)	54.0 ± 14.5	59.1 ± 13.8	55.9 ± 13.1	58.7 ± 13.7	53.4 ± 15.5	58.4 ± 14.5
Male (n)	121 (49.4%)	569 (58.9%)	42 (42.9%)	177 (59.2%)	64 (59.3%)	151 (61.1%)
Axial length (mm)	24.2 ± 1.1	25.1 ± 1.6	24.3 ± 1.5	25.0 ± 1.7	24.2 ± 1.3	24.7 ± 24.4
Average RNFL thickness (µm)	94.7 ± 8.9	71.6 ± 11.9	91.1 ± 9.5	69.7 ± 11.2	93.7 ± 7.2	73.5 ± 52.3
Average GCIPL thickness (µm)	82.1 ± 6.7	67.6 ± 10.5	80.3 ± 9.2	66.0 ± 9.2	82.3 ± 4.3	67.7 ± 9.1
HVF MD (dB)	−0.9 ± 2.6	−7.2 ± 7.6	−0.9 ± 2.4	−7.9 ± 7.0	−0.9 ± 1.2	−7.8 ± 7.2

RNFL, retinal nerve fiber layer; GCIPL, ganglion cell–inner plexiform layer; HVF, Humphrey visual field; MD, mean deviation.

**Table 2:** Comparisons among subjects from the internal validation set according to glaucoma severity.

	Early Glaucoma (MD > −6 dB) *n* = 162 (A)	Moderate Glaucoma (−6 dB ≥ MD > −12 dB) *n* = 79 (B)	Severe Glaucoma (MD ≥ −12 dB) *n* = 80 (C)	*p*	Post Hoc Analysis
Age (years)	57.1 ± 13.5	58.8 ± 14.9	61.7 ± 12.6	0.074	
Male (n)	88 (56.4%)	41 (59.4%)	48 (64.9%)	0.475	
Axial length (mm)	25.2 ± 1.7	25.2 ± 1.6	25.1 ± 1.4	0.869	
Average RNFL thickness (µm)	74.7 ± 10.4	67.9 ± 9.2	61.1 ± 8.3	<0.001	A > B > C
Average GCIPL thickness (µm)	69.3 ± 8.8	64.8 ± 7.9	60.1 ± 7.9	<0.001	A > B > C
HVF MD (dB)	−2.5 ± 1.9	−8.9 ± 1.9	−17.9 ± 4.9	<0.001	A > B > C

RNFL, retinal nerve fiber layer; GCIPL, ganglion cell–inner plexiform layer; HVF, Humphrey visual field; MD, mean deviation.

**Table 3:** Diagnostic ability of deep learning system for diagnosing glaucoma based on retinal nerve fiber layer (RNFL) and ganglion cell–inner plexiform layer (GCIPL) spectral-domain optical coherence tomography maps when testing the internal validation set.

	AUROC (95% Confidence Interval)	Sensitivity at 90% Specificity (%)	Sensitivity at 80% Specificity (%)
RNFL analysis			
Thickness map	0.987 (0.971–0.995)	97.8	98.2
Deviation map	0.974 (0.954–0.987)	93.2	97.2
Thickness map and axial length	0.975 (0.956–0.988)	93.5	95.3
GCIPL analysis			
Thickness map	0.966 (0.943–0.981)	92.5	94.6
Deviation map	0.903 (0.871–0.929)	86.6	93.1
Thickness map and axial length	0.950 (0.925–0.969)	88.8	93.7
Combination set			
RNFL deviation and GCIPL deviation map	0.979 (0.961–0.991)	94.1	97.2
RNFL deviation and GCIPL thickness map	0.963 (0.941–0.979)	91.6	96.2
RNFL thickness and GCIPL deviation map	0.952 (0.927–0.970	94.4	95.9
RNFL thickness and GCIPL thickness map	0.964 (0.942–0.980)	96.4	97.5
All 4 maps (RNFL/GCIPL thickness and deviation maps)	0.977 (0.958–0.989)	93.5	96.6
All 4 maps with axial length	0.961 (0.938–0.977)	92.8	94.0

AUROC, area under the receiver operating characteristic curve.

**Table 4:** Area under the receiver operating characteristic curve (AUROC) results of deep learning system for diagnosing glaucoma using retinal nerve fiber layer (RNFL) and ganglion cell–inner plexiform layer (GCIPL) spectral-domain optical coherence tomography maps in the internal validation dataset according to glaucoma severity.

	AUROC (95% Confidence Interval)		
	Early Glaucoma (*n* = 162)	Moderate Glaucoma (*n* = 79)	Severe Glaucoma (*n* = 80)
RNFL analysis			
Thickness map	0.974 (0.948–0.990)	0.999 (0.979–1.000)	0.999 (0.980–1.000)
Deviation map	0.956 (0.924–0.977)	0.993 (0.967–1.000)	0.993 (0.967–1.000)
Thickness map and axial length	0.951 (0.918–0.974)	0.999 (0.980–1.000)	0.999 (0.979–1.000)
GCIPL analysis			
Thickness map	0.940 (0.905–0.965)	0.991 (0.964–0.999)	0.992 (0.965–0.999)
Deviation map	0.879 (0.834–0.916)	0.919 (0.869–0.954)	0.935 (0.889–0.966)
Thickness map and axial length	0.916 (0.876–0.947)	0.981 (0.950–0.996)	0.988 (0.960–0.998)
Combination set			
RNFL deviation and GCIPL deviation map	0.965 (0.936–0.984)	0.988 (0.959–0.998)	0.999 (0.979–1.000)
RNFL deviation and GCIPL thickness map	0.947 (0.912–0.970)	0.980 (0.947–0.995)	0.981 (0.949–0.995)
RNFL thickness and GCIPL deviation map	0.939 (0.903–0.965)	0.965 (0.927–0.987)	0.965 (0.927–0.986)
RNFL thickness and GCIPL thickness map	0.952 (0.919–0.975)	0.976 (0.942–0.993)	0.976 (0.942–0.993)
All 4 maps (RNFL/GCIPL thickness and deviation maps)	0.955 (0.923–0.977)	0.999 (0.980–1.000)	0.999 (0.979–1.000)
All 4 maps with axial length	0.932 (0.895–0.959)	0.990 (0.962–0.999)	0.990 (0.963–0.999)

## Supplementary Materials

The following are available online at https://www.mdpi.com/2077-0383/9/7/2167/s1, Table S1: Glaucoma diagnostic ability of deep learning system using retinal nerve fiber layer (RNFL) and ganglion cell–inner plexiform layer (GCIPL) spectral-domain optical coherence tomography maps when testing the external validation set.
