# Supplementary material for: Development and Validation of a Deep Learning System for Diagnosing Glaucoma Using Optical Coherence Tomography
Source: J Clin Med. 2020 Jul 9;9(7):2167. doi: 10.3390/jcm9072167 (PMC7408821; doi:10.3390/jcm9072167)
Supplement: Supplementary file 1 [file jcm-09-02167-s001.pdf]

Table S1. Glaucoma diagnostic ability of deep learning system using retinal nerve fiber layer (RNFL) and ganglion cell-inner plexiform layer (GCIPL) spectral-domain optical coherence tomography maps when testing the external validation set.

	**AUROC (95% confidence interval)**	**Sensitivity at 90% specificity (%)**	**Sensitivity at 80% specificity (%)**
RNFL analysis			
Thickness map	0.981 (0.961 - 0.992)	98.4	98.4
Deviation map	0.972 (0.948 - 0.986)	93.6	96.8
Thickness map and axial length	0.974 (0.952 - 0.988)	96.4	96.5
GCIPL analysis			
Thickness map	0.982 (0.962 - 0.993)	95.2	97.6
Deviation map	0.969 (0.945 - 0.984)	92.2	97.2
Thickness map and axial length	0.970 (0.947 - 0.985)	92.4	96.0
Combination set			
RNFL deviation and GCIPL deviation map	0.985 (0.966 - 0.995)	97.2	98.2
RNFL deviation and GCIPL thickness map	0.982 (0.962 - 0.993)	95.6	96.8
RNFL thickness and GCIPL deviation map	0.981 (0.960 - 0.992)	98.0	98.4
RNFL thickness and GCIPL thickness map	0.984 (0.965 - 0.994)	98.0	98.4
All 4 maps (RNFL and GCIPL thickness, deviation maps)	0.987 (0.969 - 0.996)	97.2	98.0
All 4 maps with axial length	0.980 (0.959 - 0.992)	96.8	97.2

AUROC, area under the receiver operating characteristic curve.
